# Editorial: Celebrating Diversity and Advancements in Pediatric Cardiology—A Journey through Specialized Research

**DOI:** 10.3390/children11040455

**Published:** 2024-04-10

**Authors:** Massimo Mapelli, Paola Zagni, Irene Picciolli

**Affiliations:** 1 Centro Cardiologico Monzino, IRCCS, 20138 Milan, Italy; 2 Department of Clinical Sciences and Community Health, Cardiovascular Section, University of Milan, 20122 Milan, Italy; 3 Terapia Intensiva Neonatale, Ospedale Fatebenefratelli P.O. Macedonio Melloni, Via Macedonio Melloni 52, 20129 Milan, Italy; paola.zagni@asst-fbf-sacco.it; 4 Neonatal Intensive Care Unit, Fondazione IRCCS Ca’ Granda Ospedale Maggiore Policlinico, 20122 Milan, Italy; irene.picciolli@policlinico.mi.it

## 1. Introduction

**Figure 1:**
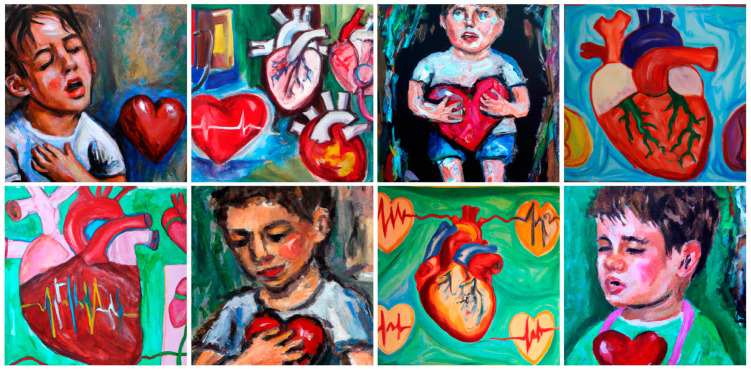
We asked generative artificial intelligence (DALL-E^®^, online version; OpenAI © 2024–2024) software to create images of children with heart disease and images representing their hearts.

The field of pediatric cardiology is as vast and diverse as the young patients it serves (Figure 1). From congenital heart diseases to acquired conditions, the complexities and challenges faced by pediatric cardiologists underscore the critical importance of specialized research aimed at improving diagnosis, treatment, and outcomes.

Pediatric cardiology stands at the forefront of medical innovation, yet amidst its advancements, critical unmet needs persist, underscoring the ongoing challenges faced in the care of young hearts. Despite significant progress in diagnostic and therapeutic modalities, several gaps in knowledge and practice continue to demand attention and resolution. One of the foremost unmet needs in pediatric cardiology lies in the realm of enhanced screening and early detection of congenital heart diseases. While advances in prenatal imaging have improved the detection of congenital abnormalities in utero [1,2], a substantial number of cases still elude detection, leading to delayed diagnosis and treatment initiation, especially in developing countries [3,4,5]. Implementing comprehensive screening protocols and leveraging emerging technologies such as fetal echocardiography and genetic testing hold promise for closing this diagnostic gap and ensuring timely interventions for infants with congenital heart diseases. Another pressing need in pediatric cardiology is the development of personalized treatment approaches tailored to the unique physiological and genetic profiles of individual patients. While broad treatment guidelines exist for common pediatric cardiac conditions, the heterogeneity of disease presentations and responses to therapy necessitate a more nuanced approach [6]. By integrating patient-specific factors such as genetic predisposition, biomarker profiles, and hemodynamic parameters, clinicians can optimize treatment strategies and improve outcomes for children with complex cardiac disorders [7,8,9]. Furthermore, there is a critical need for comprehensive long-term follow-up and transition care for pediatric cardiology patients as they transition from childhood to adolescence and adulthood. Many congenital heart diseases require lifelong surveillance and management, yet existing care models often fail to adequately address the evolving needs of patients as they age [10,11,12,13]. Establishing structured transition programs that facilitate continuity of care, empower patients and families with education and resources, and address psychosocial and vocational concerns is essential to ensuring optimal outcomes and quality of life for pediatric cardiology patients across the lifespan. Lastly, fostering innovation and research in pediatric cardiology is paramount to addressing the field’s unmet needs and driving forward progress. While significant strides have been made in understanding and treating pediatric heart conditions, numerous unanswered questions remain, particularly in areas such as regenerative medicine, targeted therapies, and disease prevention. By investing in collaborative research initiatives, harnessing cutting-edge technologies, and cultivating a culture of innovation, the pediatric cardiology community can continue to push the boundaries of knowledge and transform the landscape of pediatric cardiovascular care.

In this Special Issue of *Children* entitled “Research Progress of Pediatric Cardiology: Second Edition”, we delve into a myriad of studies showcasing the breadth and depth of pediatric cardiology research, each shedding light on different facets of this intricate field. This second volume follows the previous Special Issue [14], and taken together, these two collections provide pivotal insights in the field of pediatric cardiology.

## 2. An Overview of Published Articles

### 2.1. Exploring Cardiac Function and Cardiotoxicity in Pediatric Patients

The journey begins with investigations into cardiac function and cardiotoxicity among pediatric hemato-oncology patients. Ardelean et al. present a retrospective study evaluating the correlation of speckle-tracking echocardiography with traditional biomarkers in predicting anthracycline-induced cardiotoxicity (contribution 1). Their findings highlight the potential of novel imaging tools for early detection and prediction of cardiac injury, offering promising avenues for improved management and outcomes in this vulnerable patient population.

Continuing the exploration of cardiac function, Karki et al. (contribution 2). delve into the association between elevated copeptin levels and heart failure severity in children with cardiomyopathy. Their study underscores the prognostic value of copeptin levels in predicting adverse outcomes, providing valuable insights into risk stratification and therapeutic decision-making in pediatric cardiomyopathy.

### 2.2. Navigating the Complexities of Congenital Heart Diseases

Moving beyond acquired conditions, Kovacevic et al. shed light on the evaluation of right ventricular function in patients with propionic acidemia—a cross-sectional study that underscores the importance of comprehensive cardiac assessment in metabolic disorders (contribution 3). This study not only highlights the prevalence of right ventricular dysfunction in propionic acidemia but also emphasizes the need for tailored management strategies in this unique patient population. Similarly, a comprehensive exploration of multimodality imaging assessment of Tetralogy of Fallot (contribution 4) sheds light on the importance of cardiovascular imaging in guiding the diagnosis and long-term follow-up of patients with complex congenital heart diseases.

As we traverse the landscape of pediatric cardiology, Agati et al. draw attention to the global accessibility of cardiac surgery—a critical aspect often overlooked in discussions of healthcare disparities (contribution 5). Their review underscores the importance of addressing geographical and socioeconomic barriers to ensure equitable access to life-saving cardiac interventions across diverse populations.

### 2.3. Harnessing Advanced Imaging Modalities for Enhanced Diagnosis and Management

In their comprehensive review, Moscatelli et al. (contribution 6) provide insights into the role of cardiovascular magnetic resonance imaging across the lifespan—from fetal to adult life. Their discussion highlights the versatility and utility of CMR in the diagnosis and management of various cardiac conditions, underscoring its role as a cornerstone in modern cardiology practice.

Advancing further into the realm of imaging, Fan et al. (contribution 7) explore the predictive efficacy of pharmacological treatments in children with postural orthostatic tachycardia syndrome (POTS). Their mini-review underscores the importance of personalized medicine in optimizing therapeutic outcomes, paving the way for tailored treatment strategies in this challenging syndrome.

### 2.4. Addressing Challenges, Enhancing Care Transitions, and Embracing the Complexity and Diversity of Pediatric Cardiology

Bassareo et al. (contribution 8) tackle the critical issue of successful transition in adolescents with congenital heart disease—a vulnerable population often at risk of discontinuity in care. Through a systematic review, they highlight the importance of structured transition programs for ensuring seamless care delivery and improving long-term outcomes for adolescents with CHD.

In their exploration of stress cardiovascular imaging in the pediatric population, Moscatelli et al. illuminate the evolving landscape of diagnostic modalities in pediatric cardiology (contribution 9). Their review underscores the need for further research and standardization in stress imaging techniques, emphasizing the importance of personalized approaches in optimizing patient care.

Finally, a case series study on idiopathic pulmonary arterial hypertension (IPAH) in pediatric patients is presented (contribution 10)—a poignant reminder of the challenges faced by young patients with rare and complex cardiac conditions. Their findings underscore the need for multidisciplinary care and continued research efforts to improve outcomes in this challenging population.

## 3. Conclusions

As we reflect on the diverse array of studies presented in this Special Issue, one thing becomes abundantly clear: pediatric cardiology is a field of endless possibilities where collaboration, innovation, and hope converge to transform the lives of young patients and their families. From early detection and tailored interventions to seamless transitions and global accessibility, the journey of pediatric cardiology is marked by resilience, compassion, and a relentless pursuit of excellence. As we look ahead, let us continue to embrace the complexities and diversity of pediatric cardiology, working together to usher in a brighter future for the next generation.
